# The Effect of Hippotherapy Simulator-Assisted Therapy on Motor and Functional Outcomes in Children with Cerebral Palsy

**DOI:** 10.3390/medicina61101811

**Published:** 2025-10-09

**Authors:** Canan Günay Yazıcı, Fatih Özden, Osman Çoban, Devrim Tarakçı, Onur Aydoğdu, Zübeyir Sarı

**Affiliations:** 1 Department of Physiotherapy and Rehabilitation, Faculty of Health Sciences, Marmara University, Istanbul 34854, Türkiye; cnngnyzc@gmail.com (C.G.Y.); onur.aydogdu@marmara.edu.tr (O.A.); zubeyirsari@gmail.com (Z.S.); 2 Department of Health Care Services, Köyceğiz Vocational School of Health Services, Muğla Sıtkı Koçman University, Muğla 48800, Türkiye; 3 Department of Physiotherapy and Rehabilitation, Faculty of Health Sciences, Istanbul Üsküdar University, Istanbul 34662, Türkiye; oscoban28@gmail.com; 4 Department of Physiotherapy and Rehabilitation, Faculty of Health Sciences, İstanbul Medipol University, Istanbul 34815, Türkiye; dtarakci@medipol.edu.tr

**Keywords:** balance, cerebral palsy, hippotherapy, gait, muscle spasticity

## Abstract

*Background and Objectives*: Horse riding simulators (HRS) provide rhythmic, repetitive, and multidirectional movements analogous to horseback riding, which may facilitate postural control, balance, and functional abilities in children with cerebral palsy (CP). This study aimed to investigate the effects of the HRS application on the muscle tone of the lower extremity, gross motor function, trunk postural control, balance, gait functions, and functional independence in children with CP. *Materials and Methods*: A quasi-experimental study included 30 children with cerebral palsy (17 hemiparetic, 13 diparetic; mean age, 9.3 ± 3.2 years). All participants received Neurodevelopmental Therapy (NDT) for eight weeks, followed by eight weeks of HRS plus NDT, in a sequential design. Outcomes included the Modified Ashworth Scale (MAS), Myoton^®^PRO, Gross Motor Function Measures (GMFM)-88, Pedalo^®^ Sensamove Balance Test (Pedalo^®^ SBT), Pediatric Balance Scale (PBS), Trunk Impairment Scale (TIS), gait analysis parameters, and Functional Independence Measure (WeeFIM). Assessments were made at baseline, the 8th, and the 16th week. *Results*: At week 16, after incorporating HRS, all MAS parameters demonstrated greater improvements compared to those achieved during the first eight weeks of NDT alone (ES: 0.728–0.931, *p* < 0.05). Myoton^®^PRO measurements showed a significant reduction in gastrocnemius stiffness (ES = 0.672, *p* < 0.05) in hemiparetic children and decreases in hip adductor (ES: 0.649, *p* < 0.05) and gastrocnemius-soleus (ES: 0.766–0.865, *p* < 0.05) stiffness from week 8 to 16 in diparetic children following HRS intervention. Total scores on the GMFM-88, WeeFIM, TIS, and PBS improved significantly, with large effect sizes observed both from baseline to week 16 and from week 8 to 16 (ES: 0.771–0.886, *p* < 0.05). Additionally, Pedalo^®^ SBT scores increased following HRS intervention from baseline to week 16 (ES = 0.599–0.602, *p* < 0.05). *Conclusions*: HRS integrated with conventional NDT may improve muscle tone, motor function, balance, gait, and functional independence in children with cerebral palsy, representing a valuable adjunct to standard rehabilitation. These findings provide the first evidence that simulator-assisted interventions may benefit daily activities in children with cerebral palsy.

## 1. Introduction

Cerebral palsy (CP) is a motor disorder resulting from permanent, non-progressive damage to the immature brain that affects movement and posture [1]. Common motor impairments include primary deficits such as muscle spasticity, muscle weakness, and loss of selective motor control, which may result in secondary musculoskeletal problems, including contractures and deformities [2]. In addition, motor disorders lead to a loss of gross motor function, balance problems, a lack of postural control, and gait disturbances. All these problems limit the participation in activities of daily living (ADL) and the functional independence of children with CP [3].

There are various physiotherapeutic approaches available to reduce motor, balance, and gait disorders in children with CP. The techniques commonly used to treat these disorders focus on early interventions that promote learning and enhance neuroplasticity [4]. These include neurodevelopmental therapy (NDT), traditional therapy based on mobilizations, stretching, functional therapeutic exercises, and strengthening, as well as restraint-induced movement therapy [5,6]. All of these therapies are effective in improving gross motor function, balance, gait, and functional ability in children with CP [7].

In addition to these therapies, hippotherapy is a novel complementary treatment approach used in children with CP. Hippotherapy is an equine-assisted therapy in which a licensed therapist uses the horse’s movements to engage the sensory, neuromotor, and cognitive systems of patients with neurological disorders [8,9]. Recent literature reviews have indicated that hippotherapy yields beneficial outcomes in areas such as muscle tone, muscle symmetry, gross motor functions, postural control, balance, gait, activities of daily living, and quality of life for children with CP [5,9,10,11]. However, the benefits of hippotherapy have been widely reported, but some drawbacks hinder its use in clinical practice. Some of these risks include injury, allergic reactions, unpredictable weather, limited accessibility, high costs associated with caring for and training horses, the location and scarcity of hippotherapy centers, and the lack of licensed therapists [7,12]. Therefore, to overcome these issues, mechanical horse-riding simulators (HRS) have been developed in recent years. HRS, a type of intervention based on hippotherapy principles, imitates the movement of a real horse by producing three-dimensional movements similar to the horse walking pattern through a robotic device with a dynamic saddle [13,14]. Unlike hippotherapy, these systems offer treatment in more affordable, more accessible, safer, and controlled settings for patients with neurological impairment, such as CP [15,16,17].

Research evidence supporting the use of the HRS in the routine rehabilitation of CP is limited. Existing studies have shown the beneficial effects of the hippotherapy simulator, which can be a helpful alternative to hippotherapy, on gross motor functions, spasticity, postural control, balance, and walking function in children with CP. Although the authors emphasize that hippotherapy simulators may be a useful alternative to hippotherapy, they also state that more rigorous research is needed to prove the clinical benefits associated with this treatment [7,14,18,19,20,21]. The most recent systematic review highlighted the benefits of traditional hippotherapy and HRS applications on the gross motor functions of children with CP but noted the lack of standardized protocols in existing studies [22]. More information is required about the contribution of such therapies to the improvement of physical functions in children and their possible effects on ADL. Considering these situations, this study aimed to investigate the effects of hippotherapy simulator-assisted therapy on muscle tone, gross motor functions, trunk postural control, balance, gait parameters, and functional independence in activities of daily living in children with CP.

## 2. Materials and Methods

### 2.1. Study Design and Participants

A quasi-experimental study was conducted at the Private Dilbade Special Education and Rehabilitation Center. The guidelines of the CONsolidated Standards of Reporting Trials (CONSORT) were considered during this research [23].

**Figure 1:**
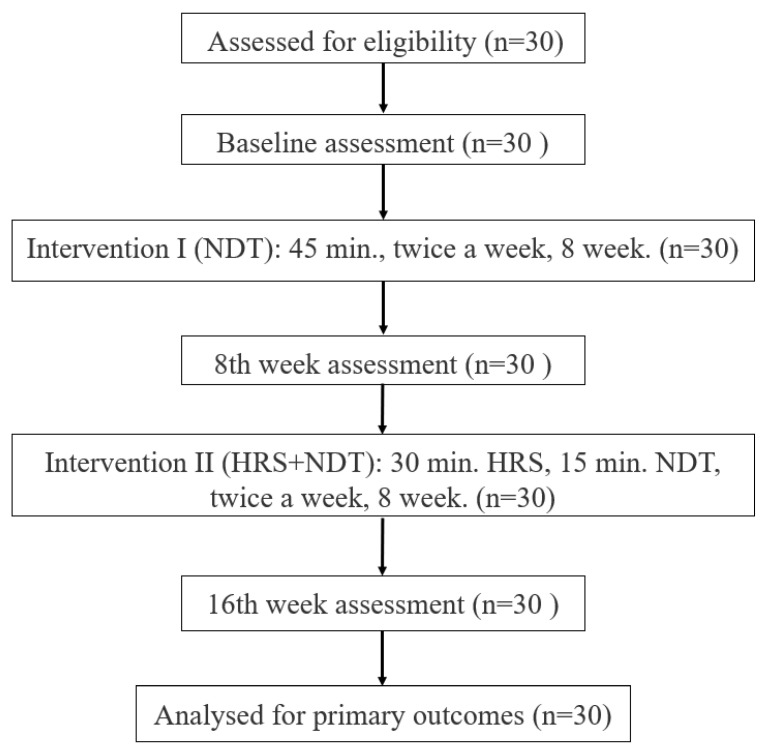
Flowchart of the study.

A total of 30 children diagnosed with spastic CP (hemiparetic and diparetic) were included in the study (Figure 1). Inclusion criteria were (1) aged 5–18 years; (2) Gross Motor Function Classification System (GMFSS) level I–III; (3) ability to sit independently; (4) ability to walk at least 10 m independently with or without an assistive device; (5) ability to cooperate with and follow verbal directions; (6) MAS adductor spasticity level 2 and below; and (7) at least 20 degrees of hip abduction. The exclusion criteria were as follows: (1) hip dislocation; (2) severe contracture or deformity that prevents study; (3) severe scoliosis (over 20 degrees); (4) acute convulsions uncontrolled by medication; (5) uncontrollable severe epileptic attacks; (6) visual and auditory problems; (7) surgical operations such as muscle relaxation, tendon lengthening, and selective dorsal rhizotomy in the last six months; (8) botulinum toxin injection in the last six months.

### 2.2. Ethical Approval

The study was conducted in accordance with the principles of ethics and the Declaration of Helsinki. The Marmara University Faculty of Medicine Clinical Research Ethics Committee approved the study protocol (No: 09.2016.478, Date: 2 September 2016). Each parent signed an informed consent form. In addition, the study was registered in ClinicalTrials.gov (NCT04378036).

### 2.3. Sample Size

The G*Power 3-based [24] post hoc power analysis was conducted to provide the power of the study sample (30 children). The lowest effect size value (0.599) of the physical function parameters over 0–16 weeks was set with the margin of error (0.05). Accordingly, the power of the study was found to be 0.83.

### 2.4. Interventions

First, all participants underwent a structured Neurodevelopmental Therapy (NDT) program, with 45 min sessions held twice a week for 8 weeks (Intervention I). After completing this initial intervention, the same group then received a different treatment combination, consisting of 15 min of NDT plus 30 min of HRS training, also twice a week for 8 weeks (Intervention II), within a sequential design.

### 2.5. Neurodevelopmental Therapy (NDT)

The NDT method is a customized treatment approach that responds to the patient’s muscle tone and movement patterns in real-time and in an individualized manner; therefore, it does not strictly adhere to a standard treatment protocol. This approach aims to achieve the most effective results by considering each individual’s unique needs and responses. In this study, a structured program was determined for each child after selecting the individual functional goals (such as improved stability in sitting and balance in standing) and targets (such as independence in ADL and walking) of the treatment. This program involved passive stretching of the lower extremity muscles (adductors, hamstrings, gastro-soleus), followed by the application of various techniques to facilitate more normal movement patterns in motor functions. The handling and facilitation principles of NDT are integrated to help improve trunk stability, balance, and gait functions. For example, assisted techniques were used to help patients sit, maintain their balance, and take steps independently. The final stage of the treatment process involved practical applications, incorporating daily living activities tailored to the children’s ages and individual abilities. This application includes play-based therapeutic activities that enhance children’s social and cognitive skills, as well as fundamental skills such as sitting, standing, and carrying objects.

### 2.6. Horse-Riding Simulator (HRS)

**Figure 2:**
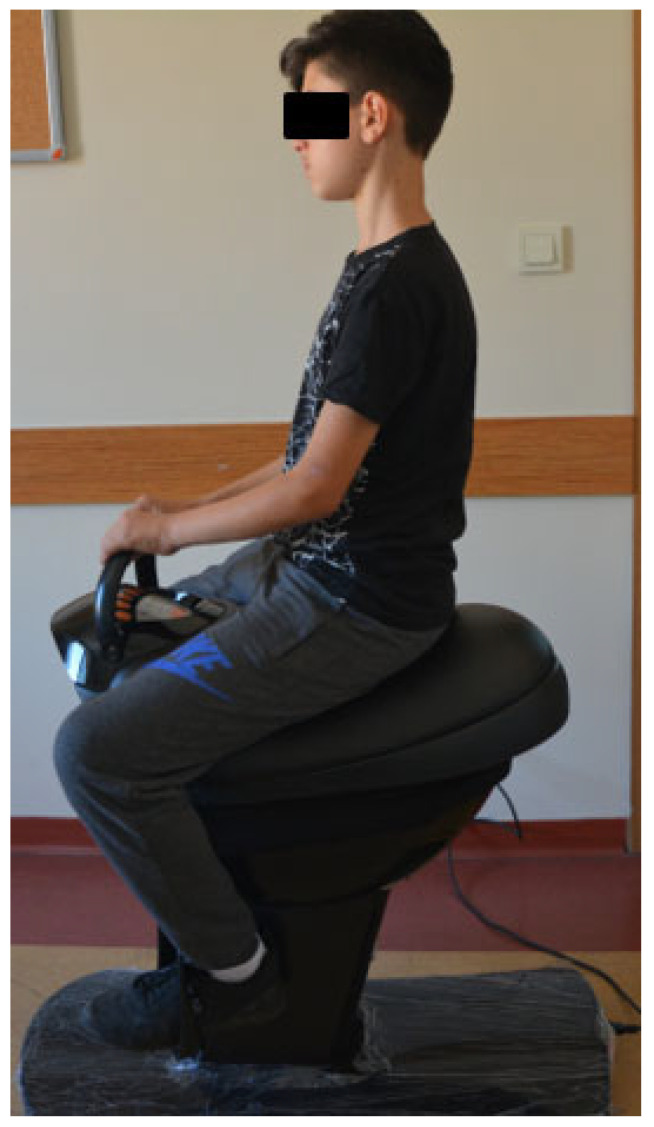
Horse Riding Simulator (HRS).

The HRS (Honjin, Anseong-si, Gyeonggi-do, Republic of Korea) (Figure 2), designed to simulate real horse movements, was utilized in this study. The system features a seat, control panel, foot pedals, and handles and is ergonomically designed for the user’s comfort and safety. It also has a workout program with four different speeds (warm-up, level 1, 2, and 3). HRS produces 8-shaped movements by oscillating forward and backward, left and right, and up and down in 3 dimensions, similar to horse movements, thus offering children a realistic horse experience.

During the treatment session, children were carefully positioned on the saddle to ensure optimal comfort and safety. Participants received instructions on how to support themselves using the handles to maintain balance and adjust their sitting position. The physiotherapist constantly encouraged children to maintain correct sitting posture during the session and provided support and stimulation to children who needed any assistance. The treatment lasted a total of 30 min, including a warm-up period (5 min), a training period (20 min), and finally a cool-down period (5 min). In the course of further treatment, depending on the children’s adaptation ability, physical response, and development, the speed was gradually increased to Level 2 and then to Level 3.

### 2.7. Outcome Measures

Participants’ clinical outcomes were evaluated at three separate time points: baseline, week 8 (Post-Intervention I), and week 16 (Post-Intervention II). The same experienced researcher performed all of these evaluations to minimize potential subjective biases.

### 2.8. Modified Ashworth Scale (MAS)

The MAS was used to measure the spasticity of the hip adductor, hamstring, gastrocnemius, and soleus muscles. Grades muscle tone from “0” to “4” with six options. Scores ranging from 0 to 5 were used for statistical analysis. The MAS level was determined by the amount of resistance shown by the spastic muscle during passive movement of the antagonist muscle [25].

### 2.9. Myoton^®^PRO

Viscoelastic properties of these muscles, such as tone, stiffness, and elasticity, were measured by the Myoton^®^PRO Digital Palpation Device (Myoton Ltd., Myoton AS, Tallinn, Estonia). Muscle stiffness (dynamic stiffness (S-N/m)), muscle tone (Oscillation Frequency (F-Hz)), and muscle flexibility (Logarithmic Decrement (D)) data were used [26]. Hip adductor muscles were measured in the supine position, and the hamstring, gastrocnemius, and soleus muscles in the prone position bilaterally. A marking point was drawn with a non-toxic pen on the palpable motor point of each muscle from the same point. The probe of the device was kept in an upright position for each muscle to be measured. The same physiotherapist took all measurements to ensure accurate palpation and measurement of the muscles.

### 2.10. Gross Motor Function Measures (GMFM)-88

Participants’ gross motor functions were evaluated with GMFM-88. In this study, the sitting (B), standing (D), and walking, running, and jumping (E) sections of the GMFM-88 were used, and the total score was obtained by calculating only the scores of these sections [27].

### 2.11. Pediatric Balance Scale (PBS)

The Pediatric Balance Scale was used to assess the participants’ balance. The scale consists of 14 different tasks to evaluate the child’s balance performance. Each item is rated from 0 to 4 (0 means “unable”, 4 means “can perform as instructed”). The maximum total score is 56, with higher scores corresponding to better balance control [28].

### 2.12. Trunk Impairment Scale (TIS)

The Trunk Impairment Scale is a tool designed to evaluate trunk performance in individuals, especially those with neurological disorders. The TIS is composed of three subscales: static sitting balance, dynamic sitting balance, and trunk coordination. For each item, a 2, 3, or 4-point ordinal scale is used. The maximal scores for the static and dynamic sitting balance and coordination subscales are 7, 10, and 6 points, respectively. The total possible score ranges from 0 (worst performance) to 23 (best performance) [29].

### 2.13. Pedalo^®^ Sensamove Balance Test (Pedalo^®^ SBT)

The Pedalo-Sensomove balance device consists of a mini-board, a circular board with hemispherical-shaped sensors placed underneath, which functions as a three-dimensional accelerometer and gyroscope with a sampling frequency of 100 Hz. This device allows the user’s movements to be recorded to obtain information about balance, reaction time, and possible body imbalances [30]. In this study, participants’ trunk control and stability were evaluated while sitting. During the sitting balance test, participants were seated on a Mini board placed on a child-friendly stool without armrests or back support, with their feet in complete contact with the ground. The screen was placed directly in front of them at eye level. Participants were instructed to focus solely on the ball displayed on the screen and maintain their balance throughout the assessment. For all tests, the maximum inclination and duration were set at 10 degrees and 30 s, respectively.

### 2.14. Gait Analysis with Win-Track

Participants’ gait parameters were measured using the Win-Track system (Medicapteurs Technology, Balma, France), a pressure platform that captures the temporal and spatial aspects of gait [31]. The participants walked on the Win-Track system in suitable outfits, without shoes, orthoses, or socks. To collect accurate data, participants performed walking trials at their own self-selected walking speed. Gait data were recorded when at least one whole step was taken and walking at a consistent pace.

### 2.15. Functional Independence Measure (WeeFIM)

The WeeFIM is an assessment tool designed to evaluate the functional abilities and independence level of children with disabilities or impairments. It consists of six subcategories: self-care, sphincter control, transfer, locomotion, communication, and social cognition. Each item is rated on a scale from 1 (total assistance required) to 7 (complete independence). The maximum score is 126 points [32].

### 2.16. Statistical Analysis

Statistical analyses were performed using SPSS (Statistical Package for the Social Sciences) Version 11.5 software. A *p*-value of less than 0.05 was considered statistically significant. Categorical variables were presented as numbers (n) and percentages (%). Continuous variables were presented as mean and standard deviation (Mean ± SD), as well as median and minimum-maximum (min–max) scores. The data did not exhibit normal distributions in normality tests conducted using the Kolmogorov–Smirnov test. Therefore, analyses were performed using non-parametric statistical methods. The “Friedman Test (analysis of variance in repeated measures)” was used to compare the outcome measures performed at baseline, after Intervention I, and after Intervention II, and to compare the changes between the evaluation results obtained. Post Hoc analyses to determine differences in time (within groups) were performed using the Wilcoxon Signed Rank Test. Bonferroni correction was made after the Friedman test. The significance value of 0.05 was divided by 3, and the new significance value was determined as *p* < 0.016. Effect sizes (ES) assessing the significance of differences within group design were calculated and expressed using the following equation:

$$r\;=\;\frac{|Z|}{\sqrt{N}}$$

*Z* = Z-score, *N* = number of participants

An effect size of r = 0.1 is considered a “small” effect, around 0.3 a “medium” effect, and 0.5 and above a “large” effect [33].

## 3. Results

### 3.1. Patient Characteristics

A total of 30 children with CP participated in the study, with a mean age of 9.3 ± 3.2 years (range: 5–15 years). Of these, 17 (56.7%) were female and 13 (43.3%) were male. Based on CP topography, 17 children (56.7%) had hemiparetic CP and 13 (43.3%) had diparetic CP. According to the GMFCS, the hemiparetic group consisted of 13 children (76.5%) at level I and four children (23.5%) at level II, while the diparetic group included five children (38.5%) at level II and eight children (61.5%) at level III. The demographic characteristics and clinical data of the children are listed in Table 1. No complications or adverse events were observed or reported during the intervention period.

### 3.2. Lower Extremity Muscle Tone

According to the MAS assessment, significant reductions in lower extremity muscle tone were observed over time in both hemiparetic and diparetic CP (*p* < 0.001). In the children with hemiparetic CP, the most pronounced decreases occurred in the hip adductors (*p* < 0.001, ES = 0.874), gastrocnemius (*p* < 0.001, ES = 0.829), and hamstrings (*p* = 0.003, ES = 0.728), with the largest effect sizes recorded after Intervention II. In children with diparetic CP on the right side, significant and large-effect reductions were noted in the hip adductors (*p* < 0.001, ES = 0.931), hamstrings (*p* < 0.001, ES = 0.930), and soleus (*p* < 0.001, ES = 0.889). On the left side, significant decreases were found in the hip adductors (*p* = 0.002, ES = 0.874), hamstrings (*p* < 0.002, ES = 0.867), and soleus (*p* = 0.002, ES = 0.842). Across both groups, Intervention II consistently produced broader and more substantial tone reductions compared to Intervention I (Table 2).

### 3.3. Myoton^®^PRO Results

Myoton^®^Pro measurements partially supported the MAS results, with significant changes most evident in the biomechanical properties of the lower extremity muscles, particularly in dynamic stiffness (S) and, to a lesser extent, oscillation frequency (F). In the hemiparetic CP, significant reductions were found in soleus oscillation frequency (F) (*p* = 0.016, ES = 0.575) and stiffness (*p* = 0.011, ES = 0.632) after Intervention I, while gastrocnemius stiffness showed a notable decrease from Intervention I to Intervention II (*p* = 0.006, ES = 0.672). No significant differences were observed in the hip adductors or hamstrings across the three time points (*p* < 0.05) (Table 3).

In the children with diparetic CP, the most prominent changes were observed following Intervention II, with significant reductions primarily in dynamic stiffness and, to a lesser extent, oscillation frequency and logarithmic decrement (D). Specifically, right hip adductors demonstrated a significant decrease in stiffness (*p* = 0.021, ES = 0.640), while left hip adductors exhibited a similar reduction (*p* = 0.014, ES = 0.649). The right hamstrings also showed a significant decline in stiffness (*p* = 0.046, ES = 0.552). Distal musculature demonstrated further pronounced changes. In the right gastrocnemius, oscillation frequency decreased significantly (*p* = 0.016, ES = 0.582), accompanied by a reduction in stiffness (*p* = 0.012, ES = 0.610) from Intervention I to Intervention II. The left gastrocnemius displayed substantial decreases in frequency (*p* = 0.002, ES = 0.850) and stiffness (*p* < 0.001, ES = 0.884) after Intervention II. The right soleus muscle exhibited significant reductions in frequency (*p* = 0.013, ES = 0.689), stiffness (*p* = 0.002, ES = 0.610–0.739), and logarithmic decrement (*p* = 0.013, ES = 0.610). Similarly, the left soleus demonstrated significant decreases in frequency (*p* = 0.015, ES = 0.650), stiffness (*p* = 0.002–0.004, ES = 0.805–0.865), and decrement (*p* = 0.013, ES = 0.691), all corresponding to large effect sizes (Table 4).

### 3.4. Gross Motor Functions

GMFM-88 in dimensions B, D, and E, as well as in total scores, showed significant increases across all time points. In dimension B, scores improved from baseline to Intervention I (*p* < 0.001, ES = 0.878) and further to Intervention II (*p* < 0.001, ES = 0.874), with a large effect size for both comparisons. Dimension D showed similar improvements (baseline–Intervention I: *p* < 0.001, ES = 0.715; baseline–Intervention II: *p* < 0.001, ES = 0.868), as did dimension E (baseline–Intervention I: *p* < 0.001, ES = 0.770; baseline–Intervention II: *p* < 0.001, ES = 0.825). The total GMFM-88 score increased significantly from baseline to Intervention I (*p* < 0.001, ES = 0.832) and to Intervention II (*p* < 0.001, ES = 0.869), with further gains between the two interventions (*p* < 0.001, ES = 0.873) (Table 5).

### 3.5. Functional Independence

The Wee-FIM subdomains and total score improved significantly. Self-care improved from baseline to Intervention I (*p* < 0.001, ES = 0.811) and further to Intervention II (*p* < 0.001, ES = 0.879). Mobility showed a similar pattern (*p* < 0.001, ES = 0.788 to 0.835). Cognition improved significantly from baseline to Intervention I (*p* < 0.001, r = 0.577) and Intervention II (*p* < 0.001, ES = 0.571), although the baseline–Intervention II comparison had a smaller effect size (ES = 0.325). The total Wee-FIM score increased significantly for all comparisons (*p* < 0.001), with large effect sizes (ES ≥ 0.848) (Table 5).

### 3.6. Trunk Postural Control

The TIS static sitting balance score improved significantly after both interventions, with a large effect observed after Intervention II (*p* < 0.001, ES = 0.833). Dynamic sitting balance showed large improvements across all comparisons (ES = 0.843–0.872). Coordination scores increased substantially, with the largest gains occurring after Intervention II (*p* < 0.001, ES = 0.895). The total TIS score increased significantly from baseline to Intervention I (*p* < 0.001, ES = 0.886) and to Intervention II (*p* < 0.001, ES = 0.827) (Table 5).

### 3.7. Balance Function

Pedalo^®^ SBT sitting balance improved significantly from baseline to Intervention I (*p* = 0.026, ES = 0.407) and further to Intervention II (*p* < 0.001, ES = 0.602). Sitting proprioception also increased significantly, with the largest effect between baseline and Intervention II (*p* < 0.001, ES = 0.599) (Table 5).

PBS scores showed marked improvements for all time points, with large effect sizes for each comparison (*p* < 0.001, ES = 0.849–0.879) (Table 5).

### 3.8. Gait Parameters

Significant improvements were observed over time in all gait parameters in children with hemiparetic and diparetic CP (*p* < 0.05), with the largest effect sizes generally occurring after Intervention II (Table 6).

In the children with hemiparetic CP, walking speed increased significantly from baseline to Intervention I (*p* < 0.001, ES = 0.878) and further to Intervention II (*p* < 0.001, ES = 0.878), with additional gains between interventions (*p* < 0.001, ES = 0.878). Cadence followed a similar pattern, improving significantly across all comparisons (*p* < 0.001, ES = 0.855–0.867). Stride length also increased significantly for all comparisons (*p* < 0.001, ES = 0.879–0.882). Stride time decreased significantly only from baseline to Intervention II (*p* = 0.016, ES = 0.612) and showed a moderate reduction from Intervention I to II (*p* = 0.052, ES = 0.471) (Table 6).

In the children with diparetic CP, gait speed improved significantly from baseline to Intervention I (*p* < 0.001, ES = 0.882) and to Intervention II (*p* < 0.001, ES = 0.882), with further improvement between interventions (*p* = 0.002, ES = 0.863). Cadence increased significantly in all comparisons (*p* < 0.001, ES = 0.882–0.883). Stride length improved from baseline to Intervention I (*p* = 0.002, ES = 0.848) and to Intervention II (*p* = 0.003, ES = 0.835), with a smaller but still significant improvement from Intervention I to II (*p* = 0.014, ES = 0.692). Stride time decreased significantly across all comparisons (*p* = 0.004, ES = 0.805–0.843) (Table 6).

Overall, both interventions resulted in notable enhancements in walking performance, with Intervention II producing the greatest improvements, particularly in walking speed, cadence, and stride length, accompanied by large effect sizes (r ≥ 0.80) in both CP subgroups (Table 6).

## 4. Discussion

The present study demonstrated that horse riding simulator-assisted training, when combined with neurodevelopmental treatment, resulted in significantly greater improvements in gross motor function, trunk postural control, balance, and gait in children with cerebral palsy compared to neurodevelopmental therapy alone. These findings suggest that incorporating HRS training into conventional rehabilitation programs may enhance functional outcomes in this population.

Management of lower extremity muscle spasticity (especially adductor spasticity) is one of the biggest challenges in the rehabilitation of children with spastic CP. Adductor muscle spasticity leads to decreased hip joint range of motion, irregular bone growth, and degenerative bone changes in children with CP [34]. Therefore, the treatment of spasticity is essential for children with CP. The lower extremity muscle spasticity (especially in the adductor muscles) of the children participating in this study, as assessed by MAS, showed a more significant improvement after Intervention II than after Intervention I. In addition, Myoton^®^PRO results showed that the tone and biomechanical properties of the lower extremity muscles of these children improved after Intervention I.

Studies investigating the effect of HRS on spasticity are limited. The results of the research by Hemachithra and colleagues indicated that the immediate impact of HRS is successful in reducing adductor spasticity [35]. Previous studies have reported that hippotherapy can reduce hip adductor muscle spasticity in children with cerebral palsy [36,37,38,39]. This decrease has been reported to result from prolonged stretching of the hip adductor muscles in the sitting position while riding a horse [36,39]. Similarly, the literature supports that sitting astride on the hippotherapy simulator provides long and slow stretching of the lower extremity muscles, including the adductor group, which always shows high muscle tension in children with spastic CP. These studies also stated that maintaining the posture against the rhythmic and repetitive movements produced by the hippotherapy simulator resulted in a reduction in muscle spasticity [13,40,41]. In this study, prolonged stretching of the lower extremity muscles for 30 min on HRS, combined with rhythmic and repetitive movements produced by HRS, can lead to decreased spasticity and improved muscle tone.

Concerning the motor function of children with CP, the primary goal of therapeutic interventions is to promote the performance of gross motor skills, which are essential components of functional mobility. Systematic review studies in the literature stated that hippotherapy is an effective treatment modality in improving gross motor functions of children with CP [9,42,43]. These studies suggest implementing hippotherapy treatment protocols for 6–10 weeks, 1–3 times a week, 30 min to 1 h a day, to improve gross motor functions in children with CP [44,45,46,47]. A similar treatment protocol was used in this study (8 weeks, 30 min HRS (+15 min NDT) twice a week). Although both groups have shown improvement in gross motor function, the HRS + NDT group demonstrated better improvement than the NDT group. Hippotherapy stimulates motor learning in children with CP, enabling the transfer of learned movement patterns to other environments [45]. Through the repetitive and rhythmic movements generated during hippotherapy, children are exposed to walking-like kinematic patterns and gradually acquire compensatory movement strategies. Similarly, HRS provides continuous, repetitive, and rhythmic stimuli that facilitate postural adjustments and motor learning. These newly acquired movement patterns are believed to support the development of gross motor functions essential for performing activities of daily living (ADLs).

Symptoms such as spasticity, movement disorder, and decreased pelvic mobility cause problems in creating automatic responses to balance and postural control, which is one of the most critical problems in children with CP [1]. In addition, sensory problems such as visual, proprioception, and tactile sensation deficiency associated with CP cause postural control and balance disturbances [10]. Many studies have proved that hippotherapy is an effective method for improving postural control and balance in children with CP [10,15,43]. Studies investigating the effects of the hippotherapy simulator on postural control and balance in children with CP are limited in the literature. Several available publications have shown that the hippotherapy simulator effectively improves sitting stability, postural control, and balance in children with CP [19,48,49]. Randomized control trials (RCTs) comparing hippotherapy and hippotherapy simulators have shown that both treatment modalities improve spinal geometry, static and dynamic balance as assessed by PBS, and sitting function in children with CP, but there is no significant difference between treatments [13,41,50]. These authors concluded that the hippotherapy simulator could be an alternative treatment to hippotherapy. RCTs comparing the hippotherapy simulator and NDT have reported that the hippotherapy simulator is more effective than NDT in improving the postural control and sitting function of children with CP [18,21]. In two studies of RCTs by Choi and Nam and colleagues, the control group received 30 min of NDT per session, four times a week for ten weeks, while the experimental group received 30 min of NDT plus 15 min of HRS. One study stated that the hippotherapy simulator, when applied in conjunction with NDT, improved the sitting balance of children with CP more than the NDT approach alone by reducing trunk imbalance, pelvic torsion, and pelvic tilt. The other study evaluated static balance using the Pedoscan system after both treatments, but no difference was found [51,52]. The results of the present study also support the literature. The HRS + NDT was more effective than NDT in developing trunk involvement and control (assessed by TIS), postural control and balance in the sitting position (measured by Pedalo SBT), and balance functions (evaluated by PBS) in children with CP who participated in this study.

In hippotherapy, improvements in postural control and balance functions in children with CP occur due to 3-dimensional rhythmic and repetitive movements of the real horse [39,50]. Making a continuous attempt to maintain the balance and posture disturbed by the horse’s rhythmic and repetitive movement while riding reveals the postural reflex and balance reactions. Thus, children learn to make postural adjustments by reducing the amount of oscillation produced by the horse and maintaining their position or central orientation; in this way, postural control and balance abilities improve [8,40,44]. The physiological effects and mechanisms of hippotherapy simulators are also similar to those of hippotherapy. The hippotherapy simulator’s continuous and rhythmic 3D reciprocal motion model creates a challenge for children with CP to maintain postural control, as in hippotherapy. Children learn to actively make anticipatory, compensatory postural adjustments and create a protective mechanism to maintain their posture against the movements of the simulator [13,21]. Multiple sensory inputs and efferent motor outputs from the CNS are continuously stimulated throughout a ride to maintain balance and posture in response to the rhythmic movement. These stimuli constantly facilitate body awareness in space, maintaining postural control and balance by eliciting unconscious muscle reactions and activating core muscles and hip adductors in the dorsal and abdominal regions of the trunk, thereby maintaining antigravity posture [12,13].

Based on the hippotherapy research literature, during riding, the horse’s movements provide continuous motor, visual, somatosensory, and vestibular inputs, which reveal the child’s postural reflexes and balance reactions. In this way, the child’s postural control and balance ability develop [40,45]. The hippotherapy simulator operates under the same principles as hippotherapy, providing sensorimotor stimulation through sensory integration between the exteroceptive, proprioceptive, and vestibular systems [53]. These stimuli are directed to the responsible areas in the cortex that generate the desired response through integrated and complementary information processing [8,45]. During a hippotherapy simulator session, cortical areas that develop with continuous stimulation of these systems can increase children’s weight-bearing, body alignment, and awareness of their center of gravity [8].

Furthermore, recent motor learning evidence has reported that at least 400–600 repetitions per session are required to improve neuroplasticity and achieve motor skill acquisition [54]. The horse generates 90 to 110 impulses per minute at a frequency of 1.5–1.8 Hz, and approximately 2700 to 3300 repetitive movements are transmitted to the rider during a 30 min hippotherapy session [55]. Similarly, hippotherapy simulators can produce 50 to 100 three-dimensional movements per minute [14,56]. In hippotherapy simulator sessions, the continuous, rhythmic, and repetitive movements made by the real horse and the simulator contribute to the development of postural control and balance by providing sufficient repetition for motor learning in children with CP. All of the factors mentioned above contribute to improving trunk stability, postural control, balance reactions, and balance functions in a sitting position. These explanations, therefore, strongly support the improvement of sitting postural control and balance function of children with CP in the present study. Many pathophysiological factors, such as spasticity, joint movement limitations, contractures, and deficiencies in postural control and balance, prevent efficient walking in children with CP [55].

In the literature, studies are limited on the effects of hippotherapy and hippotherapy simulators on the gait parameters of children with CP. Current studies show that hippotherapy improves walking speed, cadence, and stride length in children with CP [55,57]. Only one previous study has been conducted examining the effect of a hippotherapy simulation program on specific gait parameters (speed, cadence, and stride length) in children with CP. Kim and colleagues concluded that a 20 min hippotherapy simulator intervention, conducted twice a week for four weeks, increased the walking speed and stride length of children with CP [20]. Although both groups have improved gait parameters, the HRS + NDT group showed better improvement than the NDT group. This study showed that the hippotherapy simulator is an effective method for improving walking speed, stride length, and cadence in children with CP who participated in the study. The exact mechanisms through which hippotherapy and hippotherapy simulators improve gait function in children with cerebral palsy are still unknown. However, the improvement of gait function in children with cerebral palsy is believed to be due to factors such as increased movements of the pelvis, lumbar spine, and hip joints, reduction in spasticity, and improvement of balance functions [20,55]. Accordingly, in this study, the hippotherapy simulator may have contributed to improving gait parameters by regulating muscle tone and reducing spasticity, increasing sitting postural control and trunk stability, and improving balance functions in children with CP who participated in the study.

The daily life habits of children with CP are affected by activity limitations. The literature shows that hippotherapy improves self-care, mobility, and social function of children with CP and helps children participate more meaningfully in the ADL [46]. On the contrary, there is no study in the literature examining the effects of a hippotherapy simulator on ADL and the participation of children with CP. This study is the first of its kind in this regard. Its results show that the hippotherapy simulator improves children’s functional independence and participation in ADL by enhancing Wee-FIM’s self-care, mobility, and cognitive aspects. The ability to maintain posture and balance is an essential factor in performing ADLs and ensuring independent individual development. By simultaneously affecting exteroceptive, proprioceptive, and vestibular systems, a hippotherapy simulator may increase neuroplasticity and promote the modification and reorganization of the nervous system. Thus, it can improve sensorimotor learning and transform learning into a more appropriate movement model in other environments [8,39,45]. The development of postural control and balance in children with CP participating in this study, achieved through sensorimotor learning, may have increased their functional independence in various environments, including social interaction, self-care, and movement.

The development of walking functions and motor skills of children with CP who participated in this present study may have supported increased participation in ADL. On the other hand, personal and social factors affect actual performance in daily life as far as the physical environment [8,44]. Providing the child with the opportunity to use or practice communication, listening, and language skills during hippotherapy simulator sessions may have contributed to the development of the social interaction area of the Wee-FIM. In addition, a hippotherapy simulator can strengthen the motivational part of therapy by making rehabilitation more fun and engaging for children [18]. The substantial Wee-FIM gains obtained in this study demonstrate that hippotherapy simulator-assisted applications increase the child’s motivation and willingness to participate in an activity.

### Limitations

The main limitations of this present study are the small sample size and the lack of long-term follow-up of the therapies. Many of the parameters that were not statistically significant in this study did not reach sufficient statistical power. Additionally, a non-randomized quasi-experimental design with a single evaluator, which is also non-blinded, may introduce potential biases and lead to weak causal inferences.

This study included only hemiparetic and diparetic spastic-type CP and children with GMFSS levels I-III, so the results of this study cannot be generalized. We did not evaluate the effects of the hippotherapy simulator on muscle activation. Therefore, we cannot explain the impact of muscle strength and activation on improving sitting postural control, balance, and gait parameters.

From an application perspective, several issues should be considered. The cost and limited availability of simulator devices may restrict their widespread use. Therefore, while future efforts are promising, they should also focus on cost-effectiveness and accessibility in routine clinical practice.

Despite the limitations mentioned above, this study also has some significant strengths. This study is the first conducted in our country to investigate the effects of the hippotherapy simulator in children with CP. Furthermore, it is one of the first studies to investigate the effects of the hippotherapy simulator on gait parameters, spasticity, and biomechanical properties of lower extremity muscles and participation in ADL in children with CP. However, there is a need for multicenter, randomized controlled clinical studies with larger sample sizes of participants investigating the effects of a hippotherapy simulator in children with CP. Future studies should also focus on children with various CP types and GMFCS levels.

## 5. Conclusions

This present study showed that hippotherapy simulator-assisted applications have beneficial effects on lower extremity spasticity, gross motor functions, trunk and postural control in sitting position, balance, gait function, and functional independence in ADL children with CP. This study provides novel evidence that simulator-assisted interventions can improve activities of daily living, as reflected in WeeFIM scores, which have rarely been reported in previous studies. In light of all these findings, the hippotherapy simulator can be used together with NDT as an alternative therapy method for the rehabilitation of children with CP. The provision of this kind of complementary therapy added more pleasure to the rehabilitation of children with CP. It is also safe and cost-effective in a clinical setup when the real horse is unavailable. This study provides baseline data for future research and provides valuable clinical insights for physiotherapists using a hippotherapy simulator in this patient population. Future multicenter randomized controlled trials, with longer follow-up periods and the inclusion of broader CP subtypes, are warranted to confirm these results and explore their cost-effectiveness.

## Figures and Tables

**Table 1:** Demographic and clinical characteristics of children.

n = 30	Mean ± SD or n (%)
Age (year)	9.3 ± 3.2 (5–15)
Female	17 (56.7%)
Male	13 (43.3%)
Height (cm)	131.7 ± 19.1
Weight (kg)	33.6 ± 13.4
Body Mass Index (kg/m^2^)	18.5 ± 3.37
CP topography	
Hemiparetic	17 (56.7%)
Diparetic	13 (43.3%)
GMFCS Level (Hemiparetic)	
Grade 1	13 (76.5%)
Grade 2	4 (23.5%)
GMFCS Level (Diparetic)	
Grade 1	0 (0%)
Grade 2	5 (38.5%)
Grade 3	8 (61.5%)

SD: Standard Deviation, GMFCS: Gross Motor Function Classification System, CP: Cerebral Palsy.

**Table 2:** Comparison of lower extremity muscle tone according to MAS of children.

Variables	Baseline Mean ± SD Median (min–max)	Intervention I Mean ± SD Median (min–max)	Intervention II Mean ± SD Median (min–max)	*p* ^a^ T0–T1–T2	Z	*p* ^b^ T0–T1 T0–T2 T1–T2	ES T0–T1 T0–T2 T1–T2
**Hemiparetic CP (n = 17)** **(affected side)**							
Hip adductors	1.71 ± 0.98 2.00 (0–3)	1.41 ± 0.87 1.00 (0–3)	1.24 ± 0.70 1.00 (0–2)	<0.001	−2.236 −3.286 −3.606	0.025 <0.001 <0.001	0.542 0.797 ** 0.874 **
Hamstring	2.35 ± 0.60 2.00 (1–3)	2.29 ± 0.58 2.00 (1–3)	1.82 ± 0.80 2.00 (1–3)	<0.001	−1.000 −3.000 −2.828	0.317 0.003 0.005	0.243 0.728 ** 0.686 **
Gastrocnemius	2.65 ± 0.60 3.00 (1–3)	2.58 ± 0.71 3.00 (1–3)	1.76 ± 0.83 2.00 (0–3)	<0.001	−1.000 −3.419 −3.276	0.317 <0.001 <0.001	0.243 0.829 ** 0.795 **
Soleus	2.00 ± 0.79 2.00 (0–3)	1.70 ± 0.91 2.00 (0–3)	1.17 ± 0.80 1.00 (0–2)	<0.001	−2.236 −3.276 −3.000	0.025 <0.001 0.003	0.542 0.795 ** 0.728 **
**Diparetic CP (n = 13)** **(Right side)**							
Hip adductors	2.54 ± 0.66 3.00 (1–3)	2.15 ± 0.89 2.00 (0–3)	1.31 ± 0.63 1.00 (0–2)	<0.001	−2.236 −3.358 −3.317	0.025 <0.001 <0.001	0.620 0.931 ** 0.920 **
Hamstring	3.54 ± 0.51 4.00 (3–4)	2.92 ± 0.27 3.00 (2–3)	2.54 ± 0.27 3.00 (2–3)	<0.001	−2.828 −3.357 −2.236	0.005 <0.001 0.025	0.784 ** 0.930 ** 0.620
Gastrocnemius	3.31 ± 0.48 3.00 (3–4)	3.15 ± 0.37 3.00 (3–4)	2.69 ± 0.48 3.00 (2–3)	<0.001	−1.414 −2.828 −2.449	0.157 0.005 0.014	0.392 0.784 ** 0.679 **
Soleus	2.85 ± 0.37 3.00 (2–3)	2.38 ± 0.65 3.00 (1–3)	1.92 ± 0.64 2.00 (1–3)	<0.001	−2.449 −3.207 −2.449	0.014 <0.001 0.014	0.679 ** 0.889 ** 0.679 **
**Diparetic CP (n = 13)** **(Left side)**							
Hip adductors	2.77 ± 0.59 3.00 (1–3)	2.38 ± 0.76 3.00 (1–3)	1.31 ± 0.48 1.00 (1–2)	<0.001	−2.236 −3.153 −2.889	0.025 0.002 0.004	0.620 0.874 ** 0.801 **
Hamstring	3.69 ± 0.48 4.00 (3–4)	2.85 ± 0.37 3.00 (2–3)	2.69 ± 0.37 3.00 (2–3)	<0.001	−3.317 −3.127 −1.414	0.001 < 0.002 0.157	0.920 ** 0.867 ** 0.392
Gastrocnemius	3.38 ± 0.50 3.00 (3–4)	2.92 ± 0.27 3.00 (2–3)	2.69 ± 0.48 3.00 (2–3)	<0.001	−2.449 −2.460 −1.732	0.014 0.014 0.083	0.679 ** 0.682 ** 0.480
Soleus	2.85 ± 0.37 3.00 (2–3)	2.23 ± 0.43 2.00 (2–3)	1.69 ± 0.48 2.00 (1–2)	<0.001	−2.828 −3.035 −2.646	0.005 0.002 0.008	0.784 ** 0.842 ** 0.733 **

T0: Baseline; T1: Intervention I; T2: Intervention II; SD: Standard Deviation; MAS: Modified Ashworth Scale, ES: Effect Size. ^a^ Friedman test *p* < 0.05; ^b^ Wilcoxon Signed Rank test, *p* < 0.016; for ES; 0.1–0.29 indicates a small effect, 0.3–0.49 indicates a medium effect, and ≥0.5 indicates a large effect. Bold values indicate significance and medium or large effect size, ** indicates a statistically significant value.

**Table 3:** Comparison of affected side lower extremity tone by Myoton^®^Pro (hemiparetic).

Variables n = 17	Baseline Mean ± SD Median (min–max)	Intervention I Mean ± SD Median (min–max)	Intervention II Mean ± SD Median (min–max)	*p* ^a^ T0–T1–T2	Z	*p* ^b^ T0–T1 T0–T2 T1–T2	ES T0–T1 T0–T2 T1–T2
**Hip adductors**							
F (Hz)	12.24 ± 0.60 12.15 (11.15–13.50)	12.43 ± 0.72 12.40 (11.45–14.15)	12.17 ± 1.02 11.80 (10.65–14.05)	0.320	−0.829 −0.310 −1.224	0.407 0.756 0.221	0.201 0.075 0.297
S (N/m)	168.53 ± 19.13 220 (142.50–209)	171.09 ± 23.73 205 (134.50–209)	167.50 ± 29.76 203 (128–237.50)	0.943	−0.379 −0.047 −0.781	0.705 0.962 0.435	0.092 0.011 0.189
D	0.94 ± 0.14 0.98 (0.77–1.41)	0.93 ± 0.15 0.90 (0.70–1.12)	0.91 ± 0.13 0.88 (0.68–1.13)	0.943	−0.000 −0.734 −0.521	1.000 0.463 0.603	0.000 0.178 0.126
**Hamstring**							
F (Hz)	14.17 ± 1.06 14.90 (12.53–16.03)	14.16 ± 1.18 14.86 (12.47–15.82)	14.19 ± 1.11 14.10 (12.50–14.37)	0.327	−1.492 −0.024 −1.444	0.136 0.981 0.149	0.362 0.006 0.350
S (N/m)	234.73 ± 43.82 224.66 (165–329.33)	232.10 ± 48.50 222.33 (151.3–324.3)	226.29 ± 41.70 220.33 (150.6–290.3)	0.193	−1.775 −0.284 −1.538	0.076 0.776 0.124	0.430 0.069 0.373
D	1.11 ± 0.14 1.11 (0.88–1.37)	1.11 ± 0.12 1.09 (0.91–1.33)	1.08 ± 0.16 1.09 (0.77–1.40)	0.838	−0.142 −0.687 −0.758	0.887 0.492 0.449	0.034 0.167 0.184
**Gastrocnemius**							
F (Hz)	14.59 ± 1.00 14.65 (12.90–16.30)	14.80 ± 1.02 14.45 (12.88–15.30)	14.11 ± 0.92 14.38 (12.92–15.34)	0.101	−0.639 −1.681 −1.491	0.523 0.093 0.232	0.155 0.408 0.362
S (N/m)	250.79 ± 33.40 248.50 (194–300)	250.76 ± 28.76 242.50 (190.5–299.5)	232.65 ± 33.28 228.00 (186.50–301)	0.047	−0.071 −1.941 −2.770	0.943 0.052 0.006	0.017 0.471 0.672 **
D	1.10 ± 0.17 1.10 (0.81–1.46)	1.06 ± 0.18 1.01 (0.72–1.46)	1.08 ± 0.13 1.09 (0.68–1.27)	0.465	−1.302 −0.474 −0.734	0.193 0.636 0.463	0.316 0.115 0.178
**Soleus**							
F (Hz)	16.33 ± 1.43 16.40 (14.20–19.00)	15.35 ± 1.79 15.60 (11.20–18.80)	15.76 ± 1.24 15.50 (14.05–18.60)	0.011	−2.369 −1.897 −0.968	0.016 0.058 0.333	0.575 ** 0.460 0.235
S (N/m)	308.29 ± 54.17 301.00 (222–409)	279.71 ± 44.33 282.00 (200–364)	278.71 ± 41.54 274.00 (203–373)	0.004	−2.604 −2.368 −0.213	0.011 0.016 0.723	0.632 ** 0.574 ** 0.052
D	1.02 ± 0.16 0.97 (0.81–1.33)	0.97 ± 0.15 0.96 (0.73–1.28)	0.97 ± 0.13 0.95 (0.73–1.22)	0.575	−2.557 −2.368 −0.355	0.098 0.393 0.918	0.620 0.574 0.086

F: Oscillation Frequency (Muscle tone); S: Dynamic Stiffness; D: Logarithmic Decrement (Elasticity); T0: Baseline; T1: Intervention I; T2: Intervention II; SD: Standard Deviation, ES: Effect Size. ^a^ Friedman test *p* < 0.05; ^b^ Wilcoxon Signed Rank test, *p* < 0.016; for ES; 0.1–0.29 indicates a small effect, 0.3–0.49 indicates a medium effect, and ≥0.5 indicates a large effect. Bold values indicate significance and medium or large effect size, ** indicates statistically a significant value.

**Table 4:** Comparison of lower extremity tone by Myoton^®^Pro (diparetic).

Variables n = 13	Baseline Mean ± SD Median (min–max)	Intervention I Mean ± SD Median (min–max)	Intervention II Mean ± SD Median (min–max)	*p* ^a^ T0–T1–T2	Z	*p* ^b^ T0–T1 T0–T2 T1–T2	ES T0–T1 T0–T2 T1–T2
**Hip adductors (R)**							
F (Hz)	11.45 ± 0.81 11.50 (10.25–13.00)	11.51 ± 0.74 11.60 (10.40–12.65)	11.37 ± 0.58 11.50 (10.45–12.40)	0.383	−0.236 −0.455 −1.438	0.814 0.649 0.150	0.065 0.126 0.399
S (N/m)	162.31 ± 28.42 164.50 (120.5–206.0)	158.54 ± 17.72 161.00 (124–186.50)	151.42 ± 17.68 147.00 (120–178)	0.219	−0.889 −1.712 −2.308	0.374 0.087 0.021	0.247 0.475 0.640
D	1.01 ± 0.16 0.96 0.77–1.33	1.00 ± 0.16 1.02 (0.80–1.32)	1.02 ± 0.24 0.98 (0.75–1.64)	0.981	−0.784 −0.175 −0.140	0.433 0.861 0.889	0.217 0.049 0.039
**Hip adductors (L)**							
F (Hz)	11.52 ± 0.87 11.40 (10.25–12.90)	11.75 ± 0.87 12.00 (10.35–13.10)	11.41 ± 0.83 11.40 (10.20–13.10)	0.101	−0.157 −0.804 −1.893	0.875 0.421 0.058	0.044 0.223 0.525
S (N/m)	165.31 ± 31.49 171.00 (125.5–218.5)	164.19 ± 17.71 172.00 (125.50–188)	151.50 ± 23.27 149.00 (117.5–215.5)	0.050	−0.314 −1.433 −2.342	0.754 0.152 0.014	0.087 0.397 0.649 **
D	1.02 ± 0.16 0.98 (0.74–1.35)	1.00 ± 0.13 0.98 (0.84–1.32)	1.02 ± 0.20 1.01 (0.78–1.46)	0.353	−0.802 −0.350 −0.524	0.422 0.727 0.600	0.222 0.097 0.145
**Hamstring (R)**							
F (Hz)	13.91 ± 1.68 13.53 (11.4–16.13)	13.64 ± 1.59 14.00 (11.60–15.23)	13.40 ± 1.02 13.53 (11.67–15.33)	0.101	−1.177 −1.503 −1.573	0.239 0.133 0.116	0.326 0.417 0.436
S (N/m)	228.1 ± 57.4 225.66 (139–315.67)	216.15 ± 38.39 216.33 (146.67–279)	204.44 ± 34.89 204.00 (145.67–267)	0.055	−1.490 −1.712 −1.992	0.136 0.087 0.046	0.413 0.475 0.552
D	1.12 ± 0.15 1.07 (0.86–1.36)	1.10 ± 0.12 1.14 (0.92–1.31)	1.08 ± 0.16 1.06 (0.90–1.45)	0.127	−1.179 −1.398 −1.295	0.239 0.162 0.195	0.327 0.388 0.359
**Hamstring (L)**							
F (Hz)	13.86 ± 1.62 13.33 (11.8–16.33)	13.71 ± 1.38 14.10 (11.83–15.80)	13.66 ± 1.14 13.93 (11.80–15.33)	0.239	−1.060 −0.874 −1.223	0.239 0.382 0.221	0.294 0.242 0.339
S (N/m)	226.64 ± 60.58 204.33 (143–333.67)	213.51 ± 47.82 212.00 (146–292.67)	208.59 ± 35.87 221.33 (157–258.33)	0.239	−1.726 −1.503 −0.979	0.084 0.133 0.328	0.479 0.417 0.271
D	1.17 ± 0.22 1.18 (0.76–1.55)	1.15 ± 0.17 1.16 (0.91–1.44)	1.11 ± 0.15 1.08 (0.86–1.41)	0.545	−0.785 −1.154 −1.713	0.432 0.249 0.087	0.218 0.320 0.475
**Gastrocnemius (R)**							
F (Hz)	14.41 ± 1.58 13.90 (12–17.25)	14.33 ± 1.55 14.65 (11.60–17.25)	14.03 ± 1.53 13.70 (11.50–16.60)	0.025	−0.890 −1.162 −2.098	0.373 0.107 0.016	0.247 0.322 0.582 **
S (N/m)	256.96 ± 60.0 243.00 (183.50–392)	254.31 ± 54.87 205.50 (174–352)	239.19 ± 50.23 224.00 (183.50–392)	0.023	−0.942 −1.748 −2.201	0.346 0.080 0.012	0.261 0.485 0.610 **
D	1.01 ± 0.16 0.96 (0.83–1.40)	1.02 ± 0.19 0.99 (0.78–1.44)	0.97 ± 0.14 0.95 (0.76–1.27)	0.484	−1.021 −1.014 −0.979	0.307 0.310 0.327	0.283 0.281 0.271
**Gastrocnemius (L)**							
F (Hz)	14.33 ± 1.35 14.05 (12.35–16.50)	14.18 ± 1.42 14.65 (11.85–16.35)	13.60 ± 1.17 13.95 (11.85–15.40)	<0.001	−1.217 −3.113 −3.065	0.224 0.002 0.002	0.338 0.863 ** 0.850 **
S (N/m)	250.31 ± 50.63 233.50 (183–353)	238.27 ± 47.17 231.00 (168–324)	217.65 ± 47.41 224.50 (165.50–303)	<0.001	−1.804 −2.761 −3.185	0.071 0.006 < 0.001	0.500 0.766 ** 0.884 **
D	1.08 ± 0.19 1.02 (0.87–1.46)	1.01 ± 0.18 0.95 (0.76–1.38)	1.00 ± 0.19 0.94 (0.79–1.33)	0.538	−2.001 −1.255 −0.489	0.045 0.209 0.625	0.555 0.348 0.136
**Soleus (R)**							
F (Hz)	15.80 ± 2.54 15.80 (12.10–22.30)	15.46 ± 2.18 15.80 (11.90–20.70)	14.74 ± 1.63 14.80 (11.40–17.70)	<0.001	−1.780 −2.482 −2.484	0.075 0.013 0.013	0.494 0.688 ** 0.689 **
S (N/m)	306.92 ± 76.89 290.00 (196–417)	293.38 ± 66.50 280.00 (212–388)	266.85 ± 66.10 242.00 (189–388)	<0.001	−2.120 −2.667 −2.201	0.041 0.005 0.002	0.588 0.739 ** 0.610 **
D	1.03 ± 0.20 1.02 (0.85–1.62)	1.05 ± 0.33 0.97 (0.76–1.96)	0.93 ± 0.14 0.94 (0.75–1.32)	<0.001	−1.452 −2.269 −2.201	0.146 0.013 0.014	0.403 0.629 ** 0.610 **
**Soleus (L)**							
F (Hz)	15.76 ± 1.36 15.80 (12.60–17.70)	15.11 ± 1.54 15.80 (11.70–17.00)	14.47 ± 1.44 14.90 (12.20–16.10)	<0.001	−2.356 −3.112 −2.345	0.016 0.002 0.015	0.653 ** 0.863 ** 0.650 **
S (N/m)	311.15 ± 53.68 290.00 (213–378)	287.92 ± 67.43 280.00 (170–396)	251.85 ± 54.86 242.00 (183–332)	<0.001	−1.374 −3.118 −2.901	0.169 0.002 0.004	0.381 0.865 ** 0.805 **
D	1.04 ± 0.17 0.99 (0.87–1.50)	0.94 ± 0.11 0.93 (0.74–1.14)	0.94 ± 0.15 0.91 (0.62–1.17)	0.044	−2.493 −1.820 −0.771	0.013 0.069 0.441	0.691 ** 0.505 0.214

F: Oscillation Frequency (Muscle tone); S: Dynamic Stiffness; D: Logarithmic Decrement (Elasticity); T0: Baseline; T1: Intervention I; T2: Intervention II; SD: Standard Deviation; R: Right side; L: Left side, ES: Effect Size. ^a^ Friedman test *p* < 0.05; ^b^ Wilcoxon Signed Rank test, *p* < 0.016; for ES; 0.1–0.29 indicates a small effect, 0.3–0.49 indicates a medium effect, and ≥0.5 indicates a large effect. Bold values indicate significance and medium or large effect size, ** indicates a statistically significant value.

**Table 5:** Comparison of the GMFM-88, Wee-FIM, TIS, Pedalo^®^ SBT, and PBS.

Variables n = 30	Baseline Mean ± SD Median (min–max)	Intervention I Mean ± SD Median (min–max)	Intervention II Mean ± SD Median (min–max)	*p* ^a^ T0–T1–T2	Z	*p* ^b^ T0–T1 T0–T2 T1–T2	ES T0–T1 T0–T2 T1–T2
GMFM-88							
B	69.83 ± 16.62 69.16 (38.33–93.33)	75.5 ± 14.97 74.16 (43.33–96.67)	81.44 ± 12.40 78.33 (60.00–98.33)	<0.001	−4.807 −4.786 −4.731	<0.001 <0.001 <0.001	0.878 ** 0.874 ** 0.864 **
D	56.5 ± 26.20 60.25 (20.51–92.31)	61.70 ± 25.96 66.67 (23.08–94.87)	67.24 ± 25.65 73.07 (25.64–97.44)	<0.001	−3.918 −4.754 −4.713	<0.001 <0.001 <0.001	0.715 ** 0.868 ** 0.860 **
E	54.68 ± 29.08 59.02 (13.89–91.67)	58.85 ± 28.43 63.19 (16.66–94.44)	62.26 ± 28.26 65.97 (18.05–97.22)	<0.001	−4.219 −4.517 −4.715	<0.001 <0.001 <0.001	0.770 ** 0.825 ** 0.860 **
Total	60.34 ± 23.42 62.52 (25.17–91.88)	65.03 ± 22.43 67.01 (29.27–95.33)	70.32 ± 21.75 71.71 (35.12–97.66)	<0.001	−4.556 −4.762 −4.783	<0.001 <0.001 <0.001	0.832 ** 0.869 ** 0.873 **
**Wee-FIM**							
Self-care	38.70 ± 7.61 40.00 (16–51)	40.23 ± 7.13 41.00 (19–52)	42.06 ± 7.04 43.50 (21–53)	<0.001	−4.444 −4.813 −4.799	<0.001 <0.001 <0.001	0.811 ** 0.879 ** 0.876 **
Mobility	25.73 ± 6.94 26.00 (14–34)	26.66 ± 6.58 27.00 (15–35)	28.00 ± 6.06 28.50 (17–35)	<0.001	−4.315 −4.573 −4.585	<0.001 <0.001 <0.001	0.788 ** 0.835 ** 0.837 **
Cognition	29.26 ± 4.00 30.00 (22–35)	29.60 ± 4.08 30.50 (22–35)	30.03 ± 4.02 31.00 (22–35)	<0.001	−3.162 −1.782 −3.127	<0.001 <0.001 <0.001	0.577 ** 0.325 * 0.571 **
Total	93.70 ± 15.86 95.50 (61–117)	96.50 ± 15.03 101.00 (66–119)	100.10 ± 14.61 106.50 (69–121)	<0.001	−4.642 −4.789 −4.803	<0.001 <0.001 <0.001	0.848 ** 0.874 ** 0.877 **
**TIS**							
Static	4.73 ± 1.91 6.00 (2.00–7.00)	5.06 ± 1.79 6.00 (2.00–7.00)	5.66 ± 1.56 6.00 (2.00–7.00)	<0.001	−2.428 −4.563 −4.243	0.015 <0.001 <0.001	0.443 * 0.833 ** 0.775 **
Dynamic	4.63 ± 1.58 5.00 (2.00–8.00)	5.83 ± 1.41 6.00 (3.00–9.00)	7.03 ± 1.56 7.00 (4.00–10.00)	<0.001	−4.617 −4.775 −4.617	<0.001 <0.001 <0.001	0.843 ** 0.872 ** 0.843 **
Coordination	0.77 ± 0.67 1.00 (0.00–3.00)	1.23 ± 0.81 1.00 (0.00–4.00)	2.40 ± 0.89 2.00 (1.00–5.00)	<0.001	−3.742 −4.904 −5.152	<0.001 <0.001 <0.001	0.683 ** 0.895 ** 0.941 **
Total	10.13 ± 3.55 10.00 (4.00–18.00)	12.07 ± 3.47 13.00 6.00–20.00	14.80 ± 3.66 15.50 (9.00–22.00)	<0.001	−4.851 −4.529 −4.224	<0.001 <0.001 <0.001	0.886 ** 0.827 ** 0.771 **
**Pedalo^®^ SBT**							
Sitting balance	87.20 ± 9.89 91.00 (67–98)	89.27 ± 7.52 91.50 (75–98)	92.33 ± 6.76 95.00 (67–99)	<0.001	−2.228 −3.295 −3.037	0.026 <0.001 0.002	0.407 * 0.602 ** 0.555 **
Sitting proprioception	81.57 ± 13.53 87.00 (54–98)	85.07 ± 9.60 88.00 (66–98)	88.70 ± 7.23 91.50 (74–97)	<0.001	−2.093 −3.280 −3.250	0.036 <0.001 <0.001	0.382 * 0.599 ** 0.593 **
PBS	36.80 ± 13.75 43.00 (15–51)	39.37 ± 12.69 46.00 (18.52)	42.50 ± 12.06 49.00 (22–55)	<0.001	−4.649 −4.795 −4.816	<0.001 <0.001 <0.001	0.849 ** 0.875 ** 0.879 **

T0: Baseline; T1: Intervention I; T2: Intervention II; SD: Standard Deviation; Pedalo^®^ SBT; PBS: Pedalo^®^ Sensamove Balance Test; Pediatric Balance Scale, ES: Effect Size. ^a^ Friedman test *p* < 0.05; ^b^ Wilcoxon Signed Rank test, *p* < 0.016; for ES; 0.1–0.29 indicates a small effect, 0.3–0.49 indicates a medium effect, and ≥0.5 indicates a large effect. Bold values indicate significance and medium or large effect size * and ** indicates a statistically significant value.

**Table 6:** Comparison of the walking parameters of the children.

Outcome Measures	Baseline Mean ± SD Median (min–max)	Intervention I Mean ± SD Median (min–max)	Intervention II Mean ± SD Median (min–max)	*p* ^a^ T0–T1–T2	Z	*p* ^b^ T0–T1 T0–T2 T1–T2	ES T0–T1 T0–T2 T1–T2
**Hemiparetic CP (n = 17)**							
Walking speed (m/s)	0.59 ± 0.22 0.52 (0.32–1.22)	0.72 ± 0.25 0.69 (0.40–1.45)	0.83 ± 0.27 0.74 (0.49–1.62)	<0.001	−3.621 −3.621 −3.621	<0.001 <0.001 <0.001	0.878 ** 0.878 ** 0.878 **
Cadence (steps/min)	90.71 ± 12.56 90.90 (71.40–120)	104.21 ± 14.13 105.30 (69–135.50)	113.86 ± 15.59 115.40 (76–145.50)	<0.001	−3.527 −3.575 −3.528	<0.001 <0.001 <0.001	0.855 ** 0.867 ** 0.855 **
Stride length (m)	0.77 ± 0.21 0.72 (0.44–1.22)	0.83 ± 0.20 0.80 (0.49–1.28)	0.87 ± 0.21 0.85 (0.55–1.34)	<0.001	−3.628 −3.624 −3.637	<0.001 <0.001 <0.001	0.880 ** 0.879 ** 0.882 **
Stride time (s)	1.98 ± 0.25 1.95 (1.53–2.35)	1.91 ± 0.22 1.84 (1.63–2.44)	1.84 ± 0.21 1.78 (1.55–2.35)	0.013	−1.303 −2.522 −1.941	0.193 0.016 0.052	0.316 0.612 ** 0.471
**Diparetic CP (n = 13)**							
Walking speed (m/s)	0.22 ± 0.17 0.14 (0.07–0.60)	0.28 ± 0.19 0.19 (0.09–0.68)	0.33 ± 0.21 0.26 (0.11–0.75)	<0.001	−3.180 −3.180 −3.110	<0.001 <0.001 0.002	0.882 ** 0.882 ** 0.863 **
Cadence (steps/min)	50.16 ± 23.3 42.50 (28.3–95.2)	57.93 ± 23.85 57.20 (32.30–100)	64.68 ± 24.71 63.70 (35.30–105.30)	<0.001	−3.181 −3.180 −3.184	<0.001 <0.001 <0.001	0.882 ** 0.882 ** 0.883 **
Stride length (m)	0.48 ± 0.16 0.44 (0.30–0.76)	0.52 ± 0.16 0.48 (0.34–0.83)	0.56 ± 0.16 0.51 (0.37–0.86)	<0.001	−3.055 −3.011 −2.497	0.002 0.003 0.014	0.848 ** 0.835 ** 0.692 **
Stride time (s)	4.55 ± 2.02 4.82 (1.89–6.86)	4.18 ± 1,76 4.48 (1.80–6.16)	3.85 ± 1.55 3.81 (1.87–5.68)	<0.001	−3.041 −3.040 −2.901	0.002 0.002 0.004	0.843 ** 0.843 ** 0.805 **

T0: Baseline; T1: Intervention I; T2: Intervention II; SD: Standard Deviation, ES: Effect Size. ^a^ Friedman test *p* < 0.05; ^b^ Wilcoxon Signed Rank test, *p* < 0.016; for ES; 0.1–0.29 indicates a small effect, 0.3–0.49 indicates a medium effect, and ≥0.5 indicates a large effect. Bold values indicate significance and medium or large effect size, ** indicates a statistically significant value.

## Data Availability

All data generated or analyzed during this study are included in this published article.

## References

[1] Rosenbaum P., Paneth N., Leviton A., Goldstein M., Bax M., Damiano D., Dan B., Jacobsson B. (2007). A report: The definition and classification of cerebral palsy April 2006. Dev. Med. Child Neurol. Suppl..

[2] Vitrikas K., Dalton H., Breish D. (2020). Cerebral palsy: An overview. Am. Fam. Physician.

[3] Viruega H., Gaillard I., Carr J., Greenwood B., Gaviria M. (2019). Short-and mid-term improvement of postural balance after a neurorehabilitation program via hippotherapy in patients with sensorimotor impairment after cerebral palsy: A preliminary kinetic approach. Brain Sci..

[4] Novak I., Morgan C., Adde L., Blackman J., Boyd R.N., Brunstrom-Hernandez J., Cioni G., Damiano D., Darrah J., Eliasson A.C. (2017). Early, accurate diagnosis and early intervention in cerebral palsy: Advances in diagnosis and treatment. JAMA Pediatr..

[5] Novak I., Morgan C., Fahey M., Finch-Edmondson M., Galea C., Hines A., Langdon K., Namara M.M., Paton M.C., Popat H. (2020). State of the evidence traffic lights 2019: Systematic review of interventions for preventing and treating children with cerebral palsy. Curr. Neurol. Neurosci. Rep..

[6] Zanon M.A., Pacheco R.L., Latorraca C.d.O.C., Martimbianco A.L.C., Pachito D.V., Riera R. (2019). Neurodevelopmental treatment (Bobath) for children with cerebral palsy: A systematic review. J. Child Neurol..

[7] Obrero-Gaitan E., Montoro-Cardenas D., Cortes-Perez I., Osuna-Pérez M.C. (2022). Effectiveness of Mechanical Horse-Riding Simulator-Based Interventions in Patients with Cerebral Palsy—A Systematic Review and Meta-Analysis. Bioengineering.

[8] Sterba J.A. (2007). Does horseback riding therapy or therapist-directed hippotherapy rehabilitate children with cerebral palsy?. Dev. Med. Child Neurol..

[9] De Guindos-Sanchez L., Lucena-Anton D., Moral-Munoz J.A., Salazar A., Carmona-Barrientos I. (2020). The effectiveness of hippotherapy to recover gross motor function in children with cerebral palsy: A systematic review and meta-analysis. Children.

[10] Zadnikar M., Kastrin A. (2011). Effects of hippotherapy and therapeutic horseback riding on postural control or balance in children with cerebral palsy: A meta-analysis. Dev. Med. Child Neurol..

[11] Martín-Valero R., Vega-Ballón J., Pérez-Cabezas V. (2018). Benefits of hippotherapy in children with cerebral palsy: A narrative review. Eur. J. Paediatr. Neurol..

[12] Lee D.R., Lee N.G., Cha H.J., O Y.S., You S.J.H., Oh J.H., Bang H.S. (2011). The effect of robo-horseback riding therapy on spinal alignment and associated muscle size in MRI for a child with neuromuscular scoliosis: An experimenter-blind study. NeuroRehabilitation.

[13] Temcharoensuk P., Lekskulchai R., Akamanon C., Ritruechai P., Sutcharitpongsa S. (2015). Effect of horseback riding versus a dynamic and static horse riding simulator on sitting ability of children with cerebral palsy: A randomized controlled trial. J. Phys. Ther. Sci..

[14] Dominguez-Romero J.G., Molina-Aroca A., Moral-Munoz J.A., Luque-Moreno C., Lucena-Anton D. (2020). Effectiveness of mechanical horse-riding simulators on postural balance in neurological rehabilitation: Systematic review and meta-analysis. Int. J. Environ. Res. Public Health.

[15] Dewar R., Love S., Johnston L.M. (2015). Exercise interventions improve postural control in children with cerebral palsy: A systematic review. Dev. Med. Child Neurol..

[16] Kim M.J., Kim T., Oh S., Yoon B. (2018). Equine exercise in younger and older adults: Simulated versus real horseback riding. Percept. Mot. Ski..

[17] Chang H.J., Jung Y.G., Park Y.S., O S.H., Kim D.H., Kim C.W. (2021). Virtual reality-incorporated horse riding simulator to improve motor function and balance in children with cerebral palsy: A pilot study. Sensors.

[18] Borges M.B.S., Werneck M.J.d.S., Silva MdLd Gandolfi L., Pratesi R. (2011). Therapeutic effects of a horse riding simulator in children with cerebral palsy. Arq. De Neuro-Psiquiatr..

[19] Herrero P., Gómez-Trullén E.M., Asensio Á., García E., Casas R., Monserrat E., Pandyan A. (2012). Study of the therapeutic effects of a hippotherapy simulator in children with cerebral palsy: A stratified single-blind randomized controlled trial. Clin. Rehabil..

[20] Kim H.W., Nam K.S., Son S.M. (2019). Effects of virtual reality horse riding simulator training using a head-mounted display on balance and gait functions in children with cerebral palsy: A preliminary pilot study. J. Korean Phys. Ther..

[21] Chinniah H., Natarajan M., Ramanathan R., Ambrose J.W.F. (2020). Effects of horse riding simulator on sitting motor function in children with spastic cerebral palsy. Physiother. Res. Int..

[22] Ortega-Cruz A., Sánchez-Silverio V., Riquelme-Aguado V., Alonso-Perez J.L., Abuín-Porras V., Villafañe J.H. (2025). Effects of Hippotherapy and Horse-Riding Simulators on Gross Motor Function in Children with Cerebral Palsy: A Systematic Review. J. Clin. Med..

[23] Turner L., Shamseer L., Altman D.G., Weeks L., Peters J., Kober T., Dias S., Schulz K.F., Plint A.C., Moher D. (2012). Consolidated standards of reporting trials (CONSORT) and the completeness of reporting of randomised controlled trials (RCTs) published in medical journals. Cochrane Database Syst. Rev..

[24] Faul F., Erdfelder E., Lang A.-G., Buchner A. (2007). G*Power 3: A flexible statistical power analysis program for the social, behavioral, and biomedical sciences. Behav. Res. Methods.

[25] Mutlu A., Livanelioglu A., Gunel M.K. (2008). Reliability of Ashworth and Modified Ashworth scales in children with spastic cerebral palsy. BMC Musculoskelet. Disord..

[26] Wang J., Park S., Kim J. (2020). Effect of Walking with Combat Boots on the Muscle Tone and Stiffness of Lower Extremity. J. Int. Acad. Phys. Ther. Res..

[27] Palisano R.J., Hanna S.E., Rosenbaum P.L., Russell D.J., Walter S.D., Wood E.P., Raina P.S., Galuppi B.E. (2000). Validation of a model of gross motor function for children with cerebral palsy. Phys. Ther..

[28] Franjoine M.R., Gunther J.S., Taylor M.J. (2003). Pediatric balance scale: A modified version of the berg balance scale for the school-age child with mild to moderate motor impairment. Pediatr. Phys. Ther..

[29] Saether R., Helbostad J.L., Adde L., Jørgensen L., Vik T. (2013). Reliability and validity of the Trunk Impairment Scale in children and adolescents with cerebral palsy. Res. Dev. Disabil..

[30] Ahmad I., Noohu M.M., Verma S., Azharuddin M., Hussain M. (2019). Validity and Responsiveness of Balance Measures using Pedalo^®^-Sensomove Balance Device in Patients with Diabetic Peripheral Neuropathy. J. Clin. Diagn. Res..

[31] Ramachandra P., Maiya A.G., Kumar P. (2012). Test-retest reliability of the Win-Track platform in analyzing the gait parameters and plantar pressures during barefoot walking in healthy adults. Foot Ankle Spec..

[32] Tur B.S., Küçükdeveci A.A., Kutlay Ş., Yavuzer G., Elhan A.H., Tennant A. (2009). Psychometric properties of the WeeFIM in children with cerebral palsy in Turkey. Dev. Med. Child Neurol..

[33] Lakens D. (2013). Calculating and reporting effect sizes to facilitate cumulative science: A practical primer for t-tests and ANOVAs. Front. Psychol..

[34] McGibbon N.H., Benda W., Duncan B.R., Silkwood-Sherer D. (2009). Immediate and long-term effects of hippotherapy on symmetry of adductor muscle activity and functional ability in children with spastic cerebral palsy. Arch. Phys. Med. Rehabil..

[35] Hemachithra C., Meena N., Ramanathan R., Felix A. (2020). Immediate effect of horse riding simulator on adductor spasticity in children with cerebral palsy: A randomized controlled trial. Physiother. Res. Int..

[36] Benda W., McGibbon N.H., Grant K.L. (2003). Improvements in muscle symmetry in children with cerebral palsy after equine-assisted therapy (hippotherapy). J. Altern. Complement. Med..

[37] Lucena-Antón D., Rosety-Rodríguez I., Moral-Munoz J.A. (2018). Effects of a hippotherapy intervention on muscle spasticity in children with cerebral palsy: A randomized controlled trial. Complement. Ther. Clin. Pract..

[38] Alemdaroğlu E., Yanıkoğlu İ., Öken Ö., Uçan H., Ersöz M., Köseoğlu B.F., Kapıcıoğlu M.İ.S. (2016). Horseback riding therapy in addition to conventional rehabilitation program decreases spasticity in children with cerebral palsy: A small sample study. Complement. Ther. Clin. Pract..

[39] Silkwood-Sherer D., Warmbier H. (2007). Effects of hippotherapy on postural stability, in persons with multiple sclerosis: A pilot study. J. Neurol. Phys. Ther..

[40] Herrero P., Asensio Á., García E., Marco Á., Oliván B., Ibarz A., Gómez-Trullén E.M., Casas R. (2010). Study of the therapeutic effects of an advanced hippotherapy simulator in children with cerebral palsy: A randomised controlled trial. BMC Musculoskelet. Disord..

[41] Elshafey M.A. (2014). Hippotherapy simulator as alternative method for hippotherapy treatment in hemiplegic children. Int. J. Physiother. Res..

[42] Whalen C.N., Case-Smith J. (2012). Therapeutic effects of horseback riding therapy on gross motor function in children with cerebral palsy: A systematic review. Phys. Occup. Ther. Pediatr..

[43] Tseng S.-H., Chen H.-C., Tam K.-W. (2013). Systematic review and meta-analysis of the effect of equine assisted activities and therapies on gross motor outcome in children with cerebral palsy. Disabil. Rehabil..

[44] Sterba J.A., Rogers B.T., France A.P., Vokes D.A. (2002). Horseback riding in children with cerebral palsy: Effect on gross motor function. Dev. Med. Child Neurol..

[45] Casady R.L., Nichols-Larsen D.S. (2004). The effect of hippotherapy on ten children with cerebral palsy. Pediatr. Phys. Ther..

[46] Park E.S., Rha D.-W., Shin J.S., Kim S., Jung S. (2014). Effects of hippotherapy on gross motor function and functional performance of children with cerebral palsy. Yonsei Med. J..

[47] Kwon J.-Y., Chang H.J., Yi S.-H., Lee J.Y., Shin H.-Y., Kim Y.-H. (2015). Effect of hippotherapy on gross motor function in children with cerebral palsy: A randomized controlled trial. J. Altern. Complement. Med..

[48] Quint C., Toomey M. (1998). Powered saddle and pelvic mobility: An investigation into the effects on pelvic mobility of children with cerebral palsy of a powered saddle which imitates the movements of a walking horse. Physiotherapy.

[49] Kuczyński M., Słonka K. (1999). Influence of artificial saddle riding on postural stability in children with cerebral palsy. Gait Posture.

[50] Lee C.-W., Kim S.G., Na S.S. (2014). The effects of hippotherapy and a horse riding simulator on the balance of children with cerebral palsy. J. Phys. Ther. Sci..

[51] Choi H.-J., Kim K.-J., Nam K.-W. (2014). The effects of a horseback riding simulation exercise on the spinal alignment of children with cerebral palsy. J. Korean Phys. Ther..

[52] Choi H.-J., Nam K.-W. (2014). The effect of horseback riding simulator on static balance of cerebral palsy. J. Korean Phys. Ther..

[53] Park J.-H., You J.H. (2018). Innovative robotic hippotherapy improves postural muscle size and postural stability during the quiet stance and gait initiation in a child with cerebral palsy: A single case study. NeuroRehabilitation.

[54] Lang C.E., MacDonald J.R., Gnip C. (2007). Counting repetitions: An observational study of outpatient therapy for people with hemiparesis post-stroke. J. Neurol. Phys. Ther..

[55] Kwon J.-Y., Chang H.J., Lee J.Y., Ha Y., Lee P.K., Kim Y.-H. (2011). Effects of hippotherapy on gait parameters in children with bilateral spastic cerebral palsy. Arch. Phys. Med. Rehabil..

[56] Cha H.G., Lee B.J., Lee W.H. (2016). The effects of horse riding simulation exercise with blindfolding on healthy subjects’ balance and gait. J. Phys. Ther. Sci..

[57] Antunes F.N., do Pinho A.S., Kleiner A.F.R., Salazar A.P., Eltz G.D., de Oliveira Junior A.A., Cechetti F., Galli M., Pagnussat A.S. (2016). Different horse’s paces during hippotherapy on spatio-temporal parameters of gait in children with bilateral spastic cerebral palsy: A feasibility study. Res. Dev. Disabil..

